# Supplementary material for: Fins as a reliable surrogate tissue for age-related changes of telomeres and DNA methylation in gonads of a short-lived fish
Source: Biogerontology. 2025 Oct 24;26(6):195. doi: 10.1007/s10522-025-10338-4 (PMC12552393; doi:10.1007/s10522-025-10338-4)
Supplement: Supplementary file 1 — Supplementary file1 (PDF 576 KB) [file 10522_2025_10338_MOESM1_ESM.pdf]

SUPPLEMENTARY INFORMATION FOR:

Fins as a surrogate tissue for age-related changes of telomeres and DNA methylation in gonads of a short-lived fish

Milan Vrtílek[1*], Anna Kromerová[2,3], Malahat Dianat[1], Miloslava Fojtová[2,3], Dagmar Čížková[1], Jiří Fajkus[2,3*]

[1] Institute of Vertebrate Biology of the Czech Academy of Sciences, Brno, Czech Republic

[2] Mendel Centre for Plant Genomics and Proteomics, Central European Institute of Technology (CEITEC), Masaryk University, Brno, Czech Republic

[3] National Centre for Biomolecular Research, Faculty of Science, Masaryk University, Brno, Czech Republic

* corresponding authors:

Milan Vrtílek, vrtilek@ivb.cz, Institute of Vertebrate Biology of the Czech Academy of Sciences, Květná 8, 603 00 Brno, Czech Republic

Jiří Fajkus, fajkus@sci.muni.cz, Mendel Centre for Plant Genomics and Proteomics, Central European Institute of Technology (CEITEC), Masaryk University, Kamenice 753/5, 625 00 Brno, Czech Republic

Table S1. Overview of size and reproductive allotment in the experimental fish.

Table S2. The effect of changing the threshold for filtering loci with low number of reads on the results.

Figure S1. The effect of increasing filtering threshold for well-represented loci for analysis of DNA methylation.

Figure S2. Q-Q plots for the two final models testing relationship in telomere length (A) and proportion of DNA methylation (B) between fins and gonads.

Figure S3. Telomere length in fins and gonads of fish at the three AGE-samplings.

Figure S4. Ordination of DNA methylation similarity across samples based on classical multidimensional scaling coded with respect to AGE.

Appendix 1. Modified double digest restriction-site associated DNA (ddRAD) sequencing protocol for identification of DNA methylation.

Appendix 2. Parameter settings for assembling the libraries and concatenate the data using iPyrad.

Table S1. Overview of size and reproductive allotment in the experimental fish. Total body length of the fish was measured as the distance from the tip of the mouth to the end of the caudal fin. Gonado-Somatic Index (GSI) was calculated as gonad weight/dissected body weight).

**Sampling point**	**N**		**Total body length [mm]**		**Live body weight [g]**		**GSI**	
	**Female**	**Male**	**Female**	**Male**	**Female**	**Male**	**Female**	**Male**
AGE1	5	5	42.1 (±0.6)	51.0 (±0.6)	0.99 (±0.04)	1.75 (±0.07)	0.179 (±0.029)	0.006 (±0.001)
AGE2	6	5	50.3 (±1.1)	69.6 (±0.9)	1.63 (±0.12)	4.77 (±0.23)	0.184 (±0.013)	0.015 (±0.001)
AGE3	4	7	51.7 (±1.5)	68.6 (±1.5)	1.81 (±0.15)	4.28 (±0.30)	0.184 (±0.028)	0.017 (±0.001)

Table S2. The effect of changing the threshold for filtering loci with low number of reads on the results. All the RAD libraries had to have at least given number of reads ("Threshold") for the locus in question to keep it in the dataset. See also the effect of increasing the threshold from 0 to 100 on the resulting number of loci in RAD libraries and the proportion of methylation in Figure S1. The dataset based on setting the threshold to 15 (shaded row) was used in the downstream analyses. DNA methylation % was proportion of loci with zero reads in the methylation-sensitive EpiRAD library.

Threshold	Total number of loci after filtering	Mean number of reads per locus after filtering	Mean % DNA methylation	Effect of AGE	Best model of DNA methylation in gonads on fins based on AICc, R^2	Similarity ordination analysis
0	16 701	49	7.12	Fins P-value = 0.409<br>Gonads P-value = 0.242	SEX*Fin DNAm interaction, 0.825	Fins and gonads more separated
7	9 571	75	2.42	Fins P-value = 0.042<br>Gonads P-value = 0.091	SEX*Fin DNAm interaction, 0.730	Similar to 15
15	7 841	85	1.76	Fins P-value = 0.019<br>Gonads P-value = 0.073	SEX*Fin DNAm interaction, 0.689	Fig. 6
21♣	7 104	89	1.58	Fins P-value = 0.015<br>Gonads P-value = 0.082	SEX*Fin DNAm interaction, 0.713	Everything more similar than in 15
42	4 183	101	1.30	Fins P-value = 0.005<br>Gonads P-value = 0.049	SEX + Fin DNAm additive, 0.590	Everything more similar than in 15

♣ The average number of reads per locus before filtering was 21

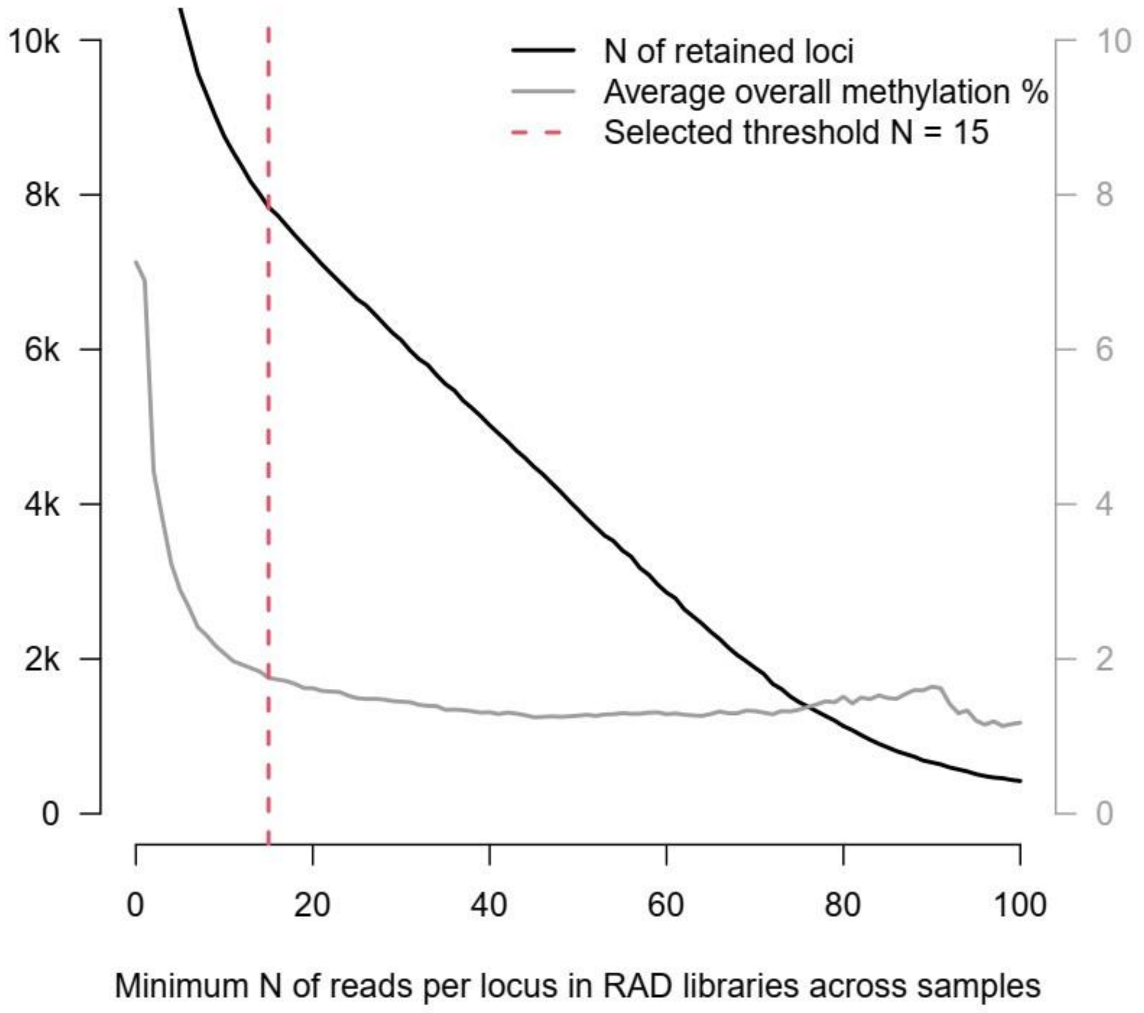

Figure S1. The effect of increasing filtering threshold for well-represented loci for analysis of DNA methylation. As the minimum read depth threshold per locus increases, the number of retained loci decreases. Additionally, the overall proportion of DNA methylation declines slightly before reaching a plateau. We decided to set the threshold to a minimum 15 reads per locus across all samples to retain sufficient number of loci for the analysis as the decline in DNA methylation proportion begins to stabilize.

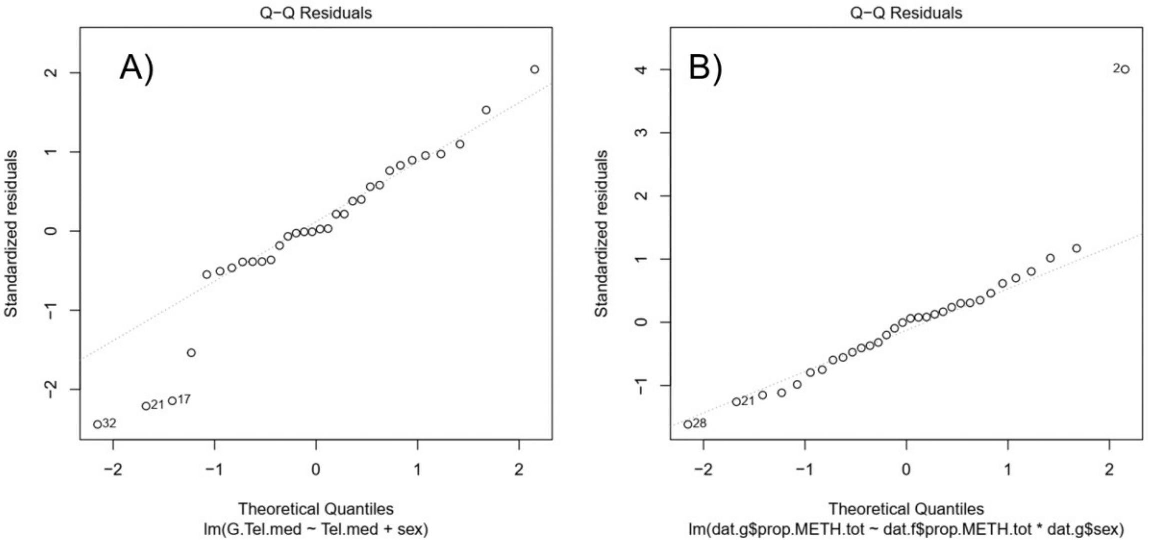

Figure S2. Q-Q plots for the two final models testing relationship in telomere length (A) and proportion of DNA methylation (B) between fins and gonads. For telomere length (A), the selected model is Telomere Length in gonad = Telomere Length in fin + SEX. For DNA methylation proportion (B), the best model includes interaction between Proportion of methylated DNA in fin and SEX. The Q-Q plots validate assumption about normal distribution of the analysed data (indicated by the dotted line). Although there is a few outliers, the overall alignment of points does not suggest a different data distribution.

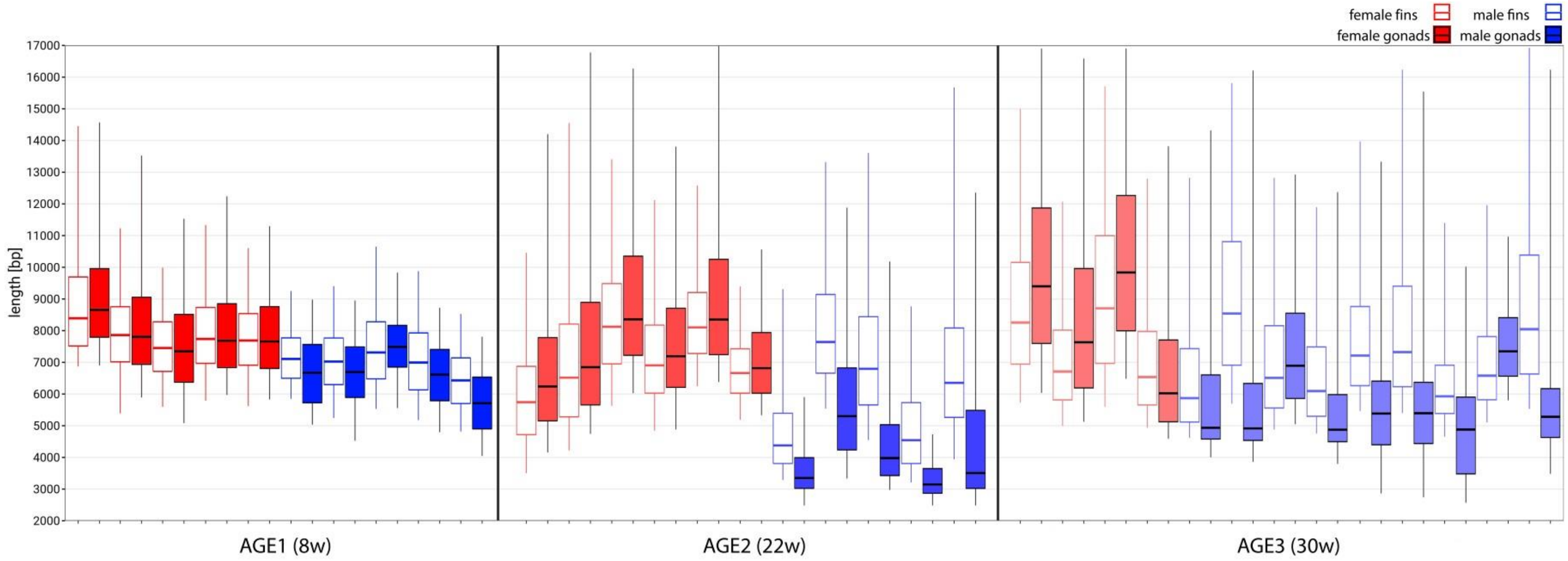

Figure S3. Telomere length in fins and gonads of fish at the three AGE-samplings. Lengths of telomeres were analysed by the TRF method, and telomerespecific hybridization signals were evaluated by the WALTER toolset (Lyčka et al., 2021). Top and bottom quartiles are separated by the median. Female, in red; male, in blue; fins, open boxes; gonads, filled boxes. Neighbouring samples (fin and gonad) were taken from the same fish.

(Lyčka, M., Peska, V., Demko, M., Spyroglou, I., Kilar, A., Fajkus, J., Fojtová, M., 2021. WALTER: an easy way to online evaluate telomere lengths from terminal restriction fragment analysis. BMC Bioinformatics 22, 1–14. https://doi.org/10.1186/s12859-021-04064-0)

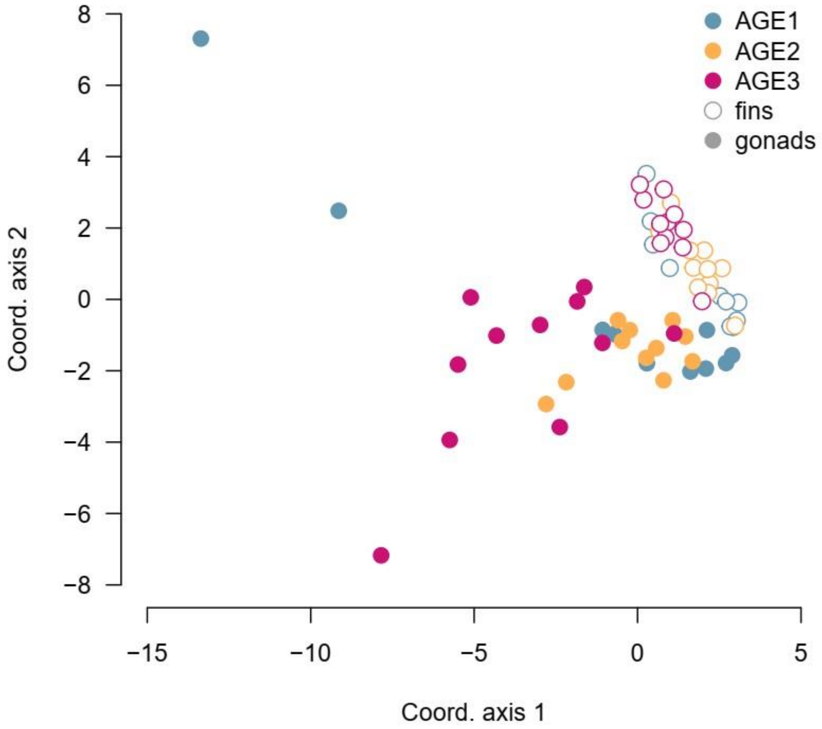

Figure S4. Ordination of DNA methylation similarity across samples based on classical multidimensional scaling coded with respect to AGE. The data is the same as for Figure 6 but colour-coded according to the AGE grouping. Coordinate axis 1 and 2 explain 9.8 % and 4.7 % of the total variation in methylation status across samples, respectively.

Appendix 1. Modified double digest restriction-site associated DNA (ddRAD-seq) protocol for identification of DNA methylation based on Peterson *et al*. (2012).

DNA extraction

We extracted DNA from the turquoise killifish fin and gonad using the cetyltrimethylammonium bromide method (Kovařík et al., 2000). The DNA integrity was verified by standard horizontal agarose gel electrophoresis, and DNA concentration was measured by the NanoDrop spectrophotometer (Thermo Fisher Scientific) and Qubit (Thermo Fisher Scientific). We aimed to obtain DNA at a concentration of 20 ng/µL; however, we also included samples with lower concentration. For ddRAD library preparation, we prioritized processing DNA samples with relatively similar concentrations together.

Adapter preparation

Oligos (Table 1) were initially eluted in TLE buffer to a final concentration of 100 µM (stock). To prepare the adapters, 50 µL of each oligo from a pair were mixed and went through the following protocol in a thermal cycler: 95°C for 1 min, followed by slow cooling of -0.1°C per second to 20°C. The i5 adapters were then diluted in TLE to a working concentration of 10 µM. Finally, they were aliquoted and stored at -20°C.

Table 1. Oligo pairs used to prepare adapters. * = PTO-Phosphorothioates, [PHO] = 5´phosphorylation. Inline barcode is in blue font.

i5-upper1	ACGACGCTCTTCCGATCTCATCC*A
i5-lower1	[PHO]AATTTGGATGAGATCGGAAGAGCGTCGTGTAGGGAAAGAGTGT
i5-upper2	ACGACGCTCTTCCGATCTAGCAAT*C
i5-lower2	[PHO]AATTGATTGCTAGATCGGAAGAGCGTCGTGTAGGGAAAGAGTGT
i7-upper1	[PHO]CGAGATCGGAAGAGCACACGTaatcc
i7-lower1	GTGACTGGAGTTCAGACGTGTGCTCTTCCGATC*T
i7-upper2	[PHO]CGTAGATCGGAAGAGCACACGTaatcc
i7-lower2	GTGACTGGAGTTCAGACGTGTGCTCTTCCGATCT*A

Protocol for ddRAD sequencing:

1) Restriction reaction:

REAGENT 1 and REAGENT 2 restriction mixes (Table 2) were incubated at 37 °C for 4 hours.

Table 2. Reagents for the restriction reactions.

REAGENT 1, non-sensitive to methylation	Per sample (µL)
H_2O	6
EcoRI-HF restriction enzyme (20,000 U/ml)	0.5
MspI restriction enzyme (20,000 U/ml)	0.5
CutSMART buffer (10× concentrated)	3
Genomic DNA	20
TOTAL VOLUME	30

REAGENT 2, sensitive to methylation	Per sample (µL)
H_2O	5.5
EcoRI-HF restriction enzyme (20,000 U/ml)	0.5

HpaII restriction enzyme (5000 U, 10U/µL)	1
CutSMART buffer (10× concentrated)	3
Genomic DNA	20
TOTAL VOLUME	30

2) Adapter ligation:

During ligation, the DNA fragments were attached to i5 and i7 adapters consisting of inline barcodes (only at i5) (Table 1). Ligation (Table 3) was done using this protocol: 23 °C for 30 minutes, 65 °C for 10 minutes, then the temperature decreased to 22 °C by 2 °C/cycle.

Table 3. Reagents for the ligation reaction.

REAGENT	Per sample (µL)
T4 DNA ligase (400 kU/ml)	0.1
Buffer (10× concentrated)	4
i5 adapter (10 µM)	0.8
i7 adapter (100 µM)	1.2
Digested DNA	17
H_2O	16.9

3) SpriSelect cleaning 1x

We followed the manufacturer's protocol for cleaning up the resulting 40 µL of DNA libraries with 40 µL of beads (1×). DNA was eluted by 20 µL of Tris buffer (10 mM); 10 µL used for PCR, 10 µL was stored as a backup.

4) PCR amplification:

Indexes were added to the libraries during the PCR amplification step (Table 4). PCR was run under these conditions: 98 °C for 45 s; 12-17 cycles (depending on DNA concentration) of 98 °C for 15 s, 60 °C for 30 s, 72 °C for 30 s; and a final extension at 72 °C for 1 min.

Table 4. Reagents for the PCR.

REAGENT	Per sample (µL)
iTru primer p5 (indexed) (10 µM)	1.25
iTru primer p7 (indexed) (10 µM)	1.25
KAPA HiFi HS Ready mix	12.5
Adapter-ligated library	10

Total volume	25

We used unique combinations (per sample) of 0.5 µM iTrue primers (Glenn et al., 2019), resulting in a dual indexed sequencing library (Table 5). Proper amplification was tested by agarose gel electrophoresis using 5 µL of PCR product.

Table 5. Indexed iTru p5 and p7 primers (Glenn et al., 2019).

PRIMER NAME	Primer sequence
iTru5_03_A	AATGATACGGCGACCACCGAGATCTACACAACACCACACACTCTTTCCCTA*C
iTru5_03_B	AATGATACGGCGACCACCGAGATCTACACTGAGCTGTACACTCTTTCCCTA*C
iTru5_03_C	AATGATACGGCGACCACCGAGATCTACACCACAGGAAACACTCTTTCCCTA*C
iTru5_03_D	AATGATACGGCGACCACCGAGATCTACACTGACAACCACACTCTTTCCCTA*C
iTru5_03_E	AATGATACGGCGACCACCGAGATCTACACTGTTCCGTACACTCTTTCCCTA*C
iTru5_03_F	AATGATACGGCGACCACCGAGATCTACACCCTAGAGAACACTCTTTCCCTA*C
iTru5_03_G	AATGATACGGCGACCACCGAGATCTACACGCATAACGACACTCTTTCCCTA*C
iTru5_03_H	AATGATACGGCGACCACCGAGATCTACACCAGTGCTTACACTCTTTCCCTA*C
iTru5_05_A	AATGATACGGCGACCACCGAGATCTACACGGTACGAAACACTCTTTCCCTA*C
iTru5_05_B	AATGATACGGCGACCACCGAGATCTACACAAGCATCGACACTCTTTCCCTA*C
iTru5_05_C	AATGATACGGCGACCACCGAGATCTACACGCCAATACACACTCTTTCCCTA*C
iTru5_05_D	AATGATACGGCGACCACCGAGATCTACACCTGTATGCACACTCTTTCCCTA*C
iTru5_05_E	AATGATACGGCGACCACCGAGATCTACACCTTAGGACACACTCTTTCCCTA*C
iTru5_05_F	AATGATACGGCGACCACCGAGATCTACACTCAGCCTTACACTCTTTCCCTA*C
iTru5_05_G	AATGATACGGCGACCACCGAGATCTACACACATGCCAACACTCTTTCCCTA*C
iTru5_05_H	AATGATACGGCGACCACCGAGATCTACACGATGGAGTACACTCTTTCCCTA*C
iTru7_201_10	CAAGCAGAAGACGGCATACGAGATTGGTATCCGTGACTGGAGTTCA*G
iTru7_201_11	CAAGCAGAAGACGGCATACGAGATGATGTCGAGTGACTGGAGTTCA*G
iTru7_204_10	CAAGCAGAAGACGGCATACGAGATGAGAAGGTGTGACTGGAGTTCA*G
iTru7_204_11	CAAGCAGAAGACGGCATACGAGATTCTTACGGGTGACTGGAGTTCA*G
iTru7_205_10	CAAGCAGAAGACGGCATACGAGATAACAGGTGGTGACTGGAGTTCA*G
iTru7_206_01	CAAGCAGAAGACGGCATACGAGATAGTCTTGGGTGACTGGAGTTCA*G
iTru7_206_09	CAAGCAGAAGACGGCATACGAGATTGTCAGTGGTGACTGGAGTTCA*G
iTru7_206_11	CAAGCAGAAGACGGCATACGAGATACAGTGACGTGACTGGAGTTCA*G
iTru7_206_12	CAAGCAGAAGACGGCATACGAGATGTTGGCATGTGACTGGAGTTCA*G
iTru7_208_03	CAAGCAGAAGACGGCATACGAGATTGCAAGACGTGACTGGAGTTCA*G
iTru7_210_03	CAAGCAGAAGACGGCATACGAGATTCACTCGAGTGACTGGAGTTCA*G
iTru7_401_08	CAAGCAGAAGACGGCATACGAGATAACCTACGGTGACTGGAGTTCA*G

iTru7_201_09	CAAGCAGAAGACGGCATACGAGATCAACCGTAGTGACTGGAGTTCA*G
iTru7_202_07	CAAGCAGAAGACGGCATACGAGATAGAGACTCGTGACTGGAGTTCA*G
iTru7_202_12	CAAGCAGAAGACGGCATACGAGATCACGCAATGTGACTGGAGTTCA*G
iTru7_203_05	CAAGCAGAAGACGGCATACGAGATTGGTTCGAGTGACTGGAGTTCA*G
iTru7_204_06	CAAGCAGAAGACGGCATACGAGATATCCGTTGGTGACTGGAGTTCA*G
iTru7_206_03	CAAGCAGAAGACGGCATACGAGATACACTACCGTGACTGGAGTTCA*G
iTru7_208_04	CAAGCAGAAGACGGCATACGAGATTGCACTTGGTGACTGGAGTTCA*G
iTru7_208_12	CAAGCAGAAGACGGCATACGAGATAGTTCGCAGTGACTGGAGTTCA*G
iTru7_210_06	CAAGCAGAAGACGGCATACGAGATTCACCTAGGTGACTGGAGTTCA*G
iTru7_401_01	CAAGCAGAAGACGGCATACGAGATTCGTGCATGTGACTGGAGTTCA*G
iTru7_401_02	CAAGCAGAAGACGGCATACGAGATATGGCGATGTGACTGGAGTTCA*G
iTru7_402_11	CAAGCAGAAGACGGCATACGAGATTGCTTGCTGTGACTGGAGTTCA*G

5) Measurement of PCR product concentration:

The approximate concentrations of PCR products were estimated using agarose gel electrophoresis according to the intensities of bands of the 100 bp ladder (Invitrogen, Thermo Fisher Scientific, USA).

6) Pooling and re-concentration of samples:

We pooled the samples equimolarly based on their concentrations, creating one subpool per gel. These subpools were subsequently pooled equimolarly into a final pool, which was then purified using SpriSelect (1.2×) and eluted in 55 µL of Tris buffer (10 mM).

We performed this step separately for the libraries derived from reagent 1 and reagent 2, and then combined the two libraries at the final stage.

7) Size selection of library pool on Pippin-prep (Sage Science, USA):

Size selection was performed following the manufacturer's instructions. The libraries were sizeselected in 306-340-374 bp.

8) Sequencing:

The final library was sent for sequencing on the Illumina NovaSeq X Plus PE150 platform at Novogene Co. Ltd., obtaining 150 bp paired-end (PE) reads.

References:

Glenn, T. C., Nilsen, R. A., Kieran, T. J., Sanders, J. G., Bayona-Vásquez, N. J., Finger, J. W., & Garcia-De Leon, F. J. (2019). Adapterama I: universal stubs and primers for 384 unique

dualindexed or 147,456 combinatorially-indexed Illumina libraries (iTru & iNext). *PeerJ*, *7*, e7755. doi: 10.7717/peerj.7755

Kovařík, A., Van Houdt, H., Holý, A., & Depicker, A. (2000). Drug-induced hypomethylation of a posttranscriptionally silenced transgene locus of tobacco leads to partial release of silencing. FEBS Letters, 467(1), 47–51. https://doi.org/10.1016/S0014-5793(00)01077-2

Peterson, B.K., Weber, J.N., Kay, E.H., Fisher, H.S., Hoekstra, H.E., 2012. Double Digest RADseq: An Inexpensive Method for De Novo SNP Discovery and Genotyping in Model and NonModel Species. *PLoS One*, 7: e37135. https://doi.org/10.1371/journal.pone.0037135

Appendix 2. Parameter settings for assembling the libraries and concatenate the data using iPyrad.

RAD libraries

```
notho_msp_PSE3                 ## [0] [assembly_name]: Assembly name. Used to name output directories for assembly steps
./                             ## [1] [project_dir]: Project dir (made in curdir if not present)
                               ## [2] [raw_fastq_path]: Location of raw non-demultiplexed fastq files
                               ## [3] [barcodes_path]: Location of barcodes file
./../*fastq                    ## [4] [sorted_fastq_path]: Location of demultiplexed/sorted fastq files reference
## [5] [assembly_method]: Assembly method (denovo, reference, denovo+reference, denovo-reference)
./../GCF_027789165.1_UI_Nfuz_MZM_1.0_genomic.fna  ## [6] [reference_sequence]: Location of reference sequence file pairddrad
## [7] [datatype]: Datatype (see docs): rad, gbs, ddrad, etc.
AATTC, CGG                     ## [8] [restriction_overhang]: Restriction overhang (cut1,) or (cut1, cut2)
5                              ## [9] [max_low_qual_bases]: Max low quality base calls (Q<20) in a read
33                             ## [10] [phred_Qscore_offset]: phred Q score offset (33 is default and very standard)
6                              ## [11] [mindepth_statistical]: Min depth for statistical base calling 6
## [12] [mindepth_majrule]: Min depth for majority-rule base calling 10000                         ##
[13] [maxdepth]: Max cluster depth within samples
0.9                            ## [14] [clust_threshold]: Clustering threshold for de novo assembly
0                              ## [15] [max_barcode_mismatch]: Max number of allowable mismatches in barcodes
2                              ## [16] [filter_adapters]: Filter for adapters/primers (1 or 2=stricter)
35                             ## [17] [filter_min_trim_len]: Min length of reads after adapter trim
2                              ## [18] [max_alleles_consens]: Max alleles per site in consensus sequences
0.05                           ## [19] [max_Ns_consens]: Max N's (uncalled bases) in consensus (R1, R2)
0.08                           ## [20] [max_Hs_consens]: Max Hs (heterozygotes) in consensus (R1, R2)
2                              ## [21] [min_samples_locus]: Min # samples per locus for output
0.2                            ## [22] [max_SNPs_locus]: Max # SNPs per locus (R1, R2)
8                              ## [23] [max_Indels_locus]: Max # of indels per locus (R1, R2)
0.5                            ## [24] [max_shared_Hs_locus]: Max # heterozygous sites per locus (R1, R2) 0,
0, 0, 0                     ## [25] [trim_reads]: Trim raw read edges (R1>, <R1, R2>, <R2) (see docs)
0, 0, 0, 0                     ## [26] [trim_loci]: Trim locus edges (see docs) (R1>, <R1, R2>, <R2)
*                              ## [27] [output_formats]: Output formats (see docs)
                               ## [28] [pop_assign_file]: Path to population assignment file
```

EpiRAD libraries

```
notho_hpa_PSE3                 ## [0] [assembly_name]: Assembly name. Used to name output directories for assembly steps
./                             ## [1] [project_dir]: Project dir (made in curdir if not present)
                               ## [2] [raw_fastq_path]: Location of raw non-demultiplexed fastq files
                               ## [3] [barcodes_path]: Location of barcodes file
```

```
./../*fastq                    ## [4] [sorted_fastq_path]: Location of demultiplexed/sorted fastq files reference
## [5] [assembly_method]: Assembly method (denovo, reference, denovo+reference, denovo-reference)
./../GCF_027789165.1_UI_Nfuz_MZM_1.0_genomic.fna  ## [6] [reference_sequence]: Location of reference sequence file pairddrad
## [7] [datatype]: Datatype (see docs): rad, gbs, ddrad, etc.
AATTC, CGG                     ## [8] [restriction_overhang]: Restriction overhang (cut1,) or (cut1, cut2)
5                              ## [9] [max_low_qual_bases]: Max low quality base calls (Q<20) in a read
33                             ## [10] [phred_Qscore_offset]: phred Q score offset (33 is default and very standard)
6                              ## [11] [mindepth_statistical]: Min depth for statistical base calling 6
## [12] [mindepth_majrule]: Min depth for majority-rule base calling
10000                          ## [13] [maxdepth]: Max cluster depth within samples
0.9                            ## [14] [clust_threshold]: Clustering threshold for de novo assembly
0                              ## [15] [max_barcode_mismatch]: Max number of allowable mismatches in barcodes
2                              ## [16] [filter_adapters]: Filter for adapters/primers (1 or 2=stricter)
35                             ## [17] [filter_min_trim_len]: Min length of reads after adapter trim
2                              ## [18] [max_alleles_consens]: Max alleles per site in consensus sequences
0.05                           ## [19] [max_Ns_consens]: Max N's (uncalled bases) in consensus (R1, R2)
0.08                           ## [20] [max_Hs_consens]: Max Hs (heterozygotes) in consensus (R1, R2)
2                              ## [21] [min_samples_locus]: Min # samples per locus for output
0.2                            ## [22] [max_SNPs_locus]: Max # SNPs per locus (R1, R2)
8                              ## [23] [max_Indels_locus]: Max # of indels per locus (R1, R2)
0.5                            ## [24] [max_shared_Hs_locus]: Max # heterozygous sites per locus (R1, R2) 0,
0, 0, 0                     ## [25] [trim_reads]: Trim raw read edges (R1>, <R1, R2>, <R2) (see docs)
0, 0, 0, 0                     ## [26] [trim_loci]: Trim locus edges (see docs) (R1>, <R1, R2>, <R2)
*                              ## [27] [output_formats]: Output formats (see docs)
                               ## [28] [pop_assign_file]: Path to population assignment file
```

Concatenation

```
notho_cat_PSE3                 ## [0] [assembly_name]: Assembly name. Used to name output directories for assembly steps
./                             ## [1] [project_dir]: Project dir (made in curdir if not present)
                               ## [2] [raw_fastq_path]: Location of raw non-demultiplexed fastq files
                               ## [3] [barcodes_path]: Location of barcodes file
./../*fastq                    ## [4] [sorted_fastq_path]: Location of demultiplexed/sorted fastq files reference
## [5] [assembly_method]: Assembly method (denovo, reference, denovo+reference, denovo-reference)
./../GCF_027789165.1_UI_Nfuz_MZM_1.0_genomic.fna  ## [6] [reference_sequence]: Location of reference sequence file pairddrad
## [7] [datatype]: Datatype (see docs): rad, gbs, ddrad, etc.
AATTC, CGG                     ## [8] [restriction_overhang]: Restriction overhang (cut1,) or (cut1, cut2)
5                              ## [9] [max_low_qual_bases]: Max low quality base calls (Q<20) in a read
33                             ## [10] [phred_Qscore_offset]: phred Q score offset (33 is default and very standard)
```

```
6                              ## [11] [mindepth_statistical]: Min depth for statistical base calling 6
## [12] [mindepth_majrule]: Min depth for majority-rule base calling 10000                          ##
[13] [maxdepth]: Max cluster depth within samples
0.9                            ## [14] [clust_threshold]: Clustering threshold for de novo assembly
0                              ## [15] [max_barcode_mismatch]: Max number of allowable mismatches in barcodes
2                              ## [16] [filter_adapters]: Filter for adapters/primers (1 or 2=stricter)
35                             ## [17] [filter_min_trim_len]: Min length of reads after adapter trim
2                              ## [18] [max_alleles_consens]: Max alleles per site in consensus sequences
0.05                           ## [19] [max_Ns_consens]: Max N's (uncalled bases) in consensus (R1, R2)
0.08                           ## [20] [max_Hs_consens]: Max Hs (heterozygotes) in consensus (R1, R2)
2                              ## [21] [min_samples_locus]: Min # samples per locus for output
0.2                            ## [22] [max_SNPs_locus]: Max # SNPs per locus (R1, R2)
8                              ## [23] [max_Indels_locus]: Max # of indels per locus (R1, R2)
0.5                            ## [24] [max_shared_Hs_locus]: Max # heterozygous sites per locus (R1, R2) 0,
0, 0, 0                     ## [25] [trim_reads]: Trim raw read edges (R1>, <R1, R2>, <R2) (see docs)
0, 0, 0, 0                     ## [26] [trim_loci]: Trim locus edges (see docs) (R1>, <R1, R2>, <R2)
*                              ## [27] [output_formats]: Output formats (see docs)
                               ## [28] [pop_assign_file]: Path to population assignment file
```
